# Effect of Ag Concentration Dispersed in HfO_x_ Thin Films on Threshold Switching

**DOI:** 10.1186/s11671-020-3258-6

**Published:** 2020-01-30

**Authors:** Won Hee Jeong, Jeong Hwan Han, Byung Joon Choi

**Affiliations:** Department of Materials Science and Engineering, Seoul National University of Science and Technology (Seoultech), Seoul, 01811 South Korea

**Keywords:** Diffusive selector, Crossbar array, Threshold switching, Co-sputtering, Electroforming process

## Abstract

A sneak path current—a current passing through a neighboring memory cell—is an inherent and inevitable problem in a crossbar array consisting of memristor memory cells. This serious problem can be alleviated by serially connecting the selector device to each memristor cell. Among the various types of selector device concepts, the diffusive selector has garnered considerable attention because of its excellent performance. This selector features volatile threshold switching (TS) using the dynamics of active metals such as Ag or Cu, which act as an electrode or dopant in the solid electrolyte. In this study, a diffusive selector based on Ag-doped HfO_x_ is fabricated using a co-sputtering system. As the Ag concentration in the HfO_x_ layer varies, different electrical properties and thereby TS characteristics are observed. The necessity of the electroforming (EF) process for the TS characteristic is determined by the proper Ag concentration in the HfO_x_ layer. This difference in the EF process can significantly affect the parameters of the TS characteristics. Therefore, an optimized doping condition is required for a diffusive selector to attain excellent selector device behavior and avoid an EF process that can eventually degrade device performance.

## Introduction

Resistance switching memory, also known as a memristor, has been extensively studied for decades as a promising candidate for next-generation nonvolatile memory. Recently, memristor devices have been applied to artificial synapses and neurons resembling their switching mechanism based on ion migration for brain-inspired computing [1–3]. Fast switching speed (< 1 ns), extreme scalability (< 2 nm), fairly good endurance (up to 10^11^ programming/erasing cycles), and three-dimensional stacking structure have been proven thus far in individual memristive systems [4–6]. In addition, image processing and pattern recognition can be enabled by building a large crossbar array (CBA) [1, 3, 7, 8].

However, a CBA structure has an inherent issue in that a sneak path current through neighboring memristor memory cells disturbs the write/read operations at the selected or half-selected cell [9–11]. To suppress the sneak path current and half-select issue, a two-terminal selector device can be serially connected to each memory cell. There are many types of selector devices with nonlinear current–voltage (*I*-*V*) characteristics being introduced, such as Schottky diodes, metal–insulator transitions (MITs), ovonic threshold switches (OTSs), tunnel barrier selectors, and diffusive selectors (also termed diffusive memristors) [9–15]. Among them, a diffusive selector based on metal species (Ag or Cu) diffusive dynamics inside the dielectrics has attracted considerable interest because of its simple structure and superior performance, such as its extremely high nonlinearity [14–22]. The diffusive selector features a volatile threshold switch (TS) based on the formation and self-rupture of metallic filaments. Various diffusive selector systems and their dynamic properties have been reported thus far; however, understanding the underlying operation mechanism remains difficult. In addition, it is necessary to establish the diffusive metal species concentration and distribution to achieve excellent TS performance because these can significantly affect the electrical conduction and transition properties.

Here, we fabricated a Pt/Ag-doped HfO_x_/Pt stack as a diffusive selector, in which Ag and HfO_x_ act as a diffusive metal dopant and dielectric material, respectively. We examined the electrical properties of the devices at different doping concentrations to relate the suitable conditions for the TS characteristic. Electroforming-needed (EF-needed) and electroforming-free (EF-free) TS characteristics were determined by the dopant concentration in the diffusive selectors. To explain the difference in the electroforming (EF) process and the subsequent TS characteristics, we performed structural and chemical analyses of the diffusive selector devices. Our study suggests the effect of dopant concentration on the TS characteristics of the diffusive selector and provides a direction to improve its performance.

## Methods

**Fig. 1:**
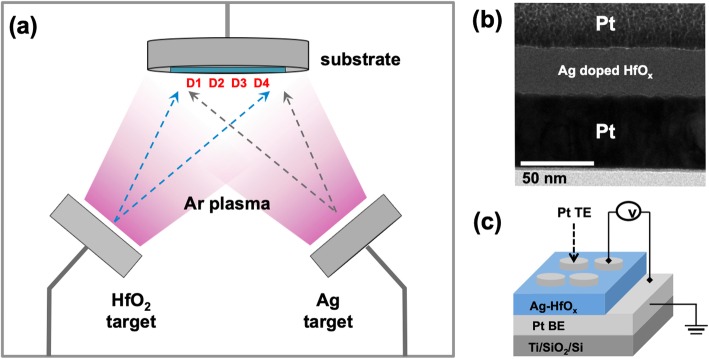
**a** Schematic diagram of a co-sputtering system. **b** Cross-sectional TEM image of the fabricated device. **c** Schematic diagram of the device with the electrical measurement system

Figure 1a shows a co-sputtering system with an Ag and HfO_2_ target. We fabricated devices placed at four positions on the substrate 0.5 cm apart from each other. The positions were near the HfO_2_ target in the order of device 1, 2, 3, and 4 (D1, D2, D3, and D4) as shown in Fig. 1a. The Ag-doped HfO_x_ switching layer of all the devices was deposited on the Pt/Ti/SiO_2_/Si substrate via co-sputtering with a sputtering power of 10 W for Ag and 150 W for HfO_2_, respectively. Before the deposition process, the vacuum chamber base pressure was evacuated up to ~ 5 × 10^6^. The switching layer was deposited for 5 min without rotating the substrate at room temperature under an Ar flow of 20 sccm to maintain the Ar plasma. The process pressure was 10 mTorr. Thereafter, the top Pt electrode was deposited on an as-deposited switching layer using a shadow mask 200 μm in diameter via e-beam evaporation. Figure 1 b and c show the cross-sectional image and schematic design of the fabricated device, respectively.

The thickness of the switching layer deposited via co-sputtering was measured using an ellipsometer (FS-1, Film-Sense, USA). Rutherford backscattering spectrometry (RBS; 6SDH-2, NEC, USA) and X-ray fluorescence spectrometry (XRF; ARL, Thermo Fisher Scientific, USA) were performed to analyze the atomic composition of the Ag-doped HfO_x_ layer. To evaluate the device electrical properties, a semiconductor parameter analyzer (SPA; HP-4155A, Agilent, USA) was used at room temperature. All the measurements were conducted by applying a bias to the top electrode while the bottom electrode was grounded as shown in Fig. 1c. The surface of the devices was analyzed using a field emission scanning electron microscope (FE-SEM; JSM-6700F, JEOL, Japan) and an atomic force microscopy (AFM; XE-100, Park system, Korea). The cross-sectional samples of the devices were prepared using a focused ion beam (FIB; Quanta 3D FEG, FEI, Netherlands) process and were observed using a high-resolution transmission electron microscope (HR-TEM: JEM-2100F, JEOL, Japan). The chemical composition was analyzed using energy-dispersive X-ray spectroscopy (EDS).

## Results and Discussion

We simultaneously fabricated four Ag-doped HfO_x_ devices for diffusive selectors during a single process. During the deposition process, the substrate was not rotated to confirm the effect of the doping concentration on the electrical properties. Table 1 shows the thickness, Ag:Hf cation ratio, and root mean square (RMS) roughness of D1, D2, D3, and D4, respectively. The Ag composition in the switching layer was expressed as a cation ratio using an amount of Ag and Hf obtained from the XRF analysis. As shown, each device has a different thickness and Ag concentration. With an increase in the distance from the HfO_2_ target, the switching layer thickness decreased while the Ag composition in the oxide layer increased. With increasing Ag concentration, the RMS roughness values slightly increased (AFM images are shown in Additional file 1: Figure S1).

**Table 1:** Thickness, cation atomic ratio, and roughness of the fabricated devices

	D1	D2	D3	D4
Thickness (nm)	35.3	27.3	24	18.8
Cation ratio (%)	7	16	39	58
RMS roughness (nm)	0.29	1.59	2.15	3.55

**Fig. 2:**
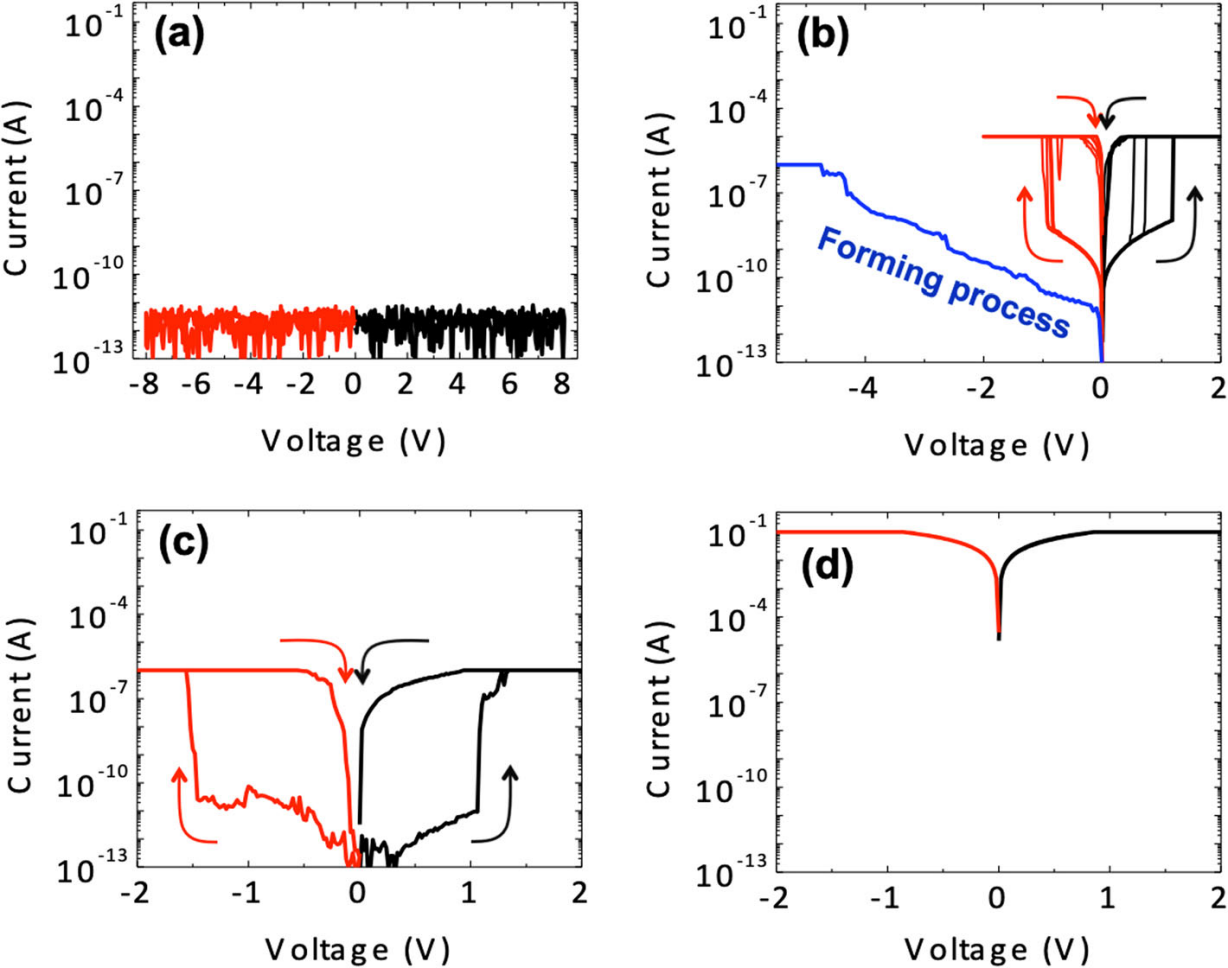
Electrical properties of the fabricated devices. **a** *I-V* curve of D1 (highly insulating state). **b** *I-V* curve of D2, showing the EF process and subsequent TS behaviors. **c** *I-V* curve of D3, showing TS behavior without the EF process. **d** *I-V* curve of D4 (conducting state)

Direct current (DC)–voltage (*I*-*V*) characteristics measured from all devices are shown in Fig. 2a–d. D1 (35.3 nm, 7% Ag) was initially in a highly insulating state, and no threshold switching was observed during the DC measurements (Fig. 1a) because of the insufficient Ag concentration to produce a conductive filament despite the thick switching layer. In contrast, D2 (27.3 nm, 16% Ag) showed a threshold switching from a high-resistance state (HRS) to a low-resistance state (LRS) following the electroforming (EF) process in the pristine state to produce an operable device as shown in Fig. 1b. During the EF process, the current increased from a low level to reach a compliance current (*I*_cc_) at the voltage of − 4.3 V. Thereafter, D2 continuously showed TS behavior at a lower operating voltage than the forming voltage in both bias polarities. Similarly, D3 (24 nm, 39% Ag) showed a typical bidirectional TS behavior; however, the EF process was not needed in the pristine state of D3. In other words, D3 presents EF-free TS behavior. In contrast, D4 (18.8 nm, 58 Ag%) was initially in a highly conducting state, probably because of Ag percolation within the thin HfO_x_ layer given the high Ag concentration.

Surface morphology and grain size changed with increasing Ag concentration. As previously noted, with increasing Ag concentration, the RMS roughness values increased as shown in Table 1. The grain size was also assessed using an SEM (Additional file 1: Figure S2). An increase in grain size was observed as the Ag concentration increased. However, in the case of D2 and D3 that show disparate TS characteristics, the difference in the surface roughness and grain size was quite small. Nevertheless, there was a considerable difference in their electric properties in terms of the EF process and following TS characteristics. Thus, we further compared the TS characteristics of D2 and D3 as follows.

**Fig. 3:**
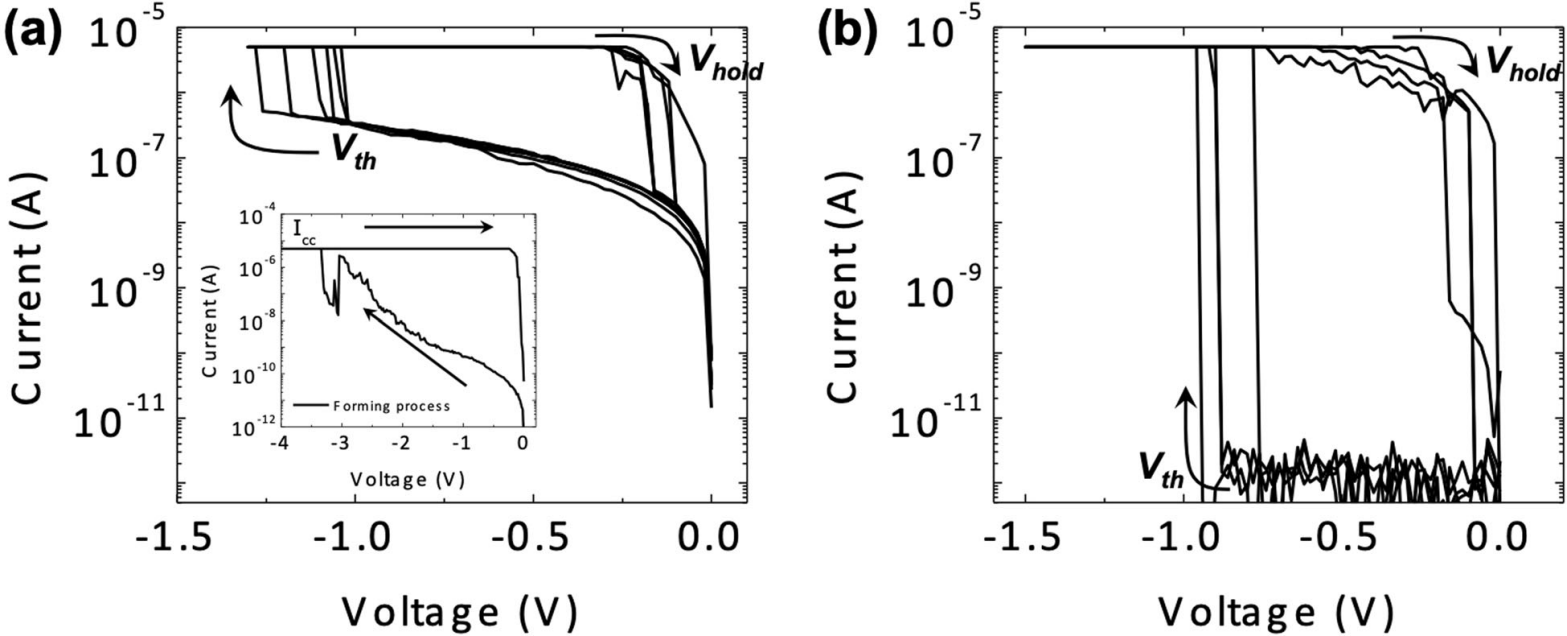
Comparison of TS characteristics in D2 and D3. **a** TS behavior with an increased OFF current following the EF process. The inset figure shows the EF process in an as-deposited device. **b** TS behavior without the EF process maintaining a low OFF current and high *NL*

Figure 3 a and b show the repeatable TS behavior observed in D2 and D3 via DC measurement. For comparison, only the TS characteristics at the negative bias are shown in the figures. Both devices initially showed several pA current levels at − 0.1 V under the detection limit. TS behavior in D2 was evident after the EF process at a forming voltage of ~ − 3.5 V, while a compliance current (*I*_cc_) of 5 μA was set for the device to prevent a hard breakdown. Following the EF process, the device showed typical TS behavior as shown in Fig. 3a. When the applied voltage exceeded the threshold voltage (*V*_th_) of ~ − 1.1 V, the current suddenly reached an *I*_cc_ of 5 μA; the device switched to the ON state from the OFF state. However, the device ON state recovered to the OFF state as the applied voltage decreased to less than the hold voltage (*V*_hold_). Although the device returned to the OFF state, a higher OFF current was observed than that of the device before EF.

D3 also showed typical TS characteristics as shown in Fig. 3b. However, the EF process was not required to induce TS behavior in the device in the pristine state. The current reached an *I*_cc_ of 5 μA at a *V*_th_ of ~ − 0.8 V, which is the ON state, and then spontaneously returned to the initial OFF state at a *V*_th_ less than ~ − 0.2 V. The subsequent D3 *I*-*V* loops were similar to the first *I*-*V* loops. Moreover, the device consistently showed a low OFF current at a low operating voltage compared with that of D2. Additionally, the OFF state current density in D3 still remained less than that of D2; the difference was approximately 10^5^ A/cm^2^. Consequently, it was confirmed that the difference in the Ag concentration in the HfO_x_ layer determined the necessity of the EF process, and in turn, the TS characteristics dramatically changed.

To realize a one selector–one resistive memory (1S1R), a selector requires a low OFF current to suppress the leakage current and a high ON current corresponding to the reset current of the resistive memory [21, 22]. To fulfill such *I*-*V* nonlinear characteristics, we confirmed the nonlinearity (*NL*) and selectivity (*S*) of our devices and those of TS selectors previously reported to evaluate their performance as selectors [14, 18, 21–23]. Here, we define *NL* and *S* using Eqs. (1) and (2), respectively, as follows:

1 $$ NL=\frac{I_{V_{\mathrm{th}}}}{I_{\frac{1}{2}{V}_{\mathrm{th}}}} $$

2 $$ S=\frac{I_{\mathrm{ON}}}{I_{\mathrm{OFF}}} $$

*NL* is defined as the ratio of the current at *V*_th_ and one-half of *V*_th_. In the half-bias scheme in the crossbar array, *V*_th_ is applied to the targeting cell while one-half of *V*_th_ is applied to the neighboring half-selected cells. Therefore, *NL* is critical to prevent the crossbar array malfunctioning during the program/read operations. In contrast, *S* is the ratio of the current in the ON state and OFF state at *V*_th_, representing the performance of the TS-based selector. Both definitions are widely used for the comparison of selector device performance. Thus, large *NL* and *S* values are required for the 1S1R operation to effectively suppress the sneak path current.

Various switching parameters including *NL* and *S* in our devices and the TS selectors in the literature are shown in Table 2. In the case of D2, an increase in the OFF current causes a significant decrease in *NL* and *S*. In contrast, the D3 OFF current is sufficiently low such that a greater than 10^6^ *NL* and *S* were acquired. However, D2 and D3 could only show a TS characteristic at a low *I*_cc_ (< 10 μA) because TS transitioned to memory switching at a higher *I*_cc_. It is well known that most TS selector devices using Ag filament are subject to a transition of nonvolatile memory switching at an *I*_cc_ greater than 10–100 μA [23–26]. When *I*_cc_ is higher than 10–100 μA, a robust and stable metal filament is formed that is difficult to spontaneously rupture compared with the thin and unstable filament formed at a lower *I*_cc_ [26, 27]. Therefore, various methods, such as a multilayer structure (Ag/TaO_x_/TaO_y_/TaO_x_/Ag) and Ag nanodots templated in the dielectric, have been suggested to obtain reliable TS characteristics at a higher *I*_cc_ [21, 22].

**Table 2:** Recent reported selectors using Ag-filament TS including our devices

	*NL*	*S*	|*V*_th_|, |*V*_hold_|	Max. *I* (*I*_cc_)
D2 (our work)	40	45	1–1.2 V, ~ 0.2 V	10 μA
D3 (our work)	8.2 × 10^6^	4.2 × 10^6^	0.7–0.9 V, ~ 0.2 V	10 μA
Ag/a-Si:H/Pt [23]	~ 10^6^	~ 10^6^	0.7–0.9 V, 0.4–0.5 V	10 μA
Pd/Ag/HfO_2_/Ag/Pd [18]	~ 10^8^	~ 10^8^	0.5 V, 0.1 V	100 μA
Pt/MgO:Ag/Pt [14]	~ 5 × 10^3^	~ 5 × 10^3^	0.3 V, ~ 0 V	100 μA
Pt:SiO_x_N_y_:Ag/Pt [14]	~ 10^5^	~ 10^5^	0.3 V, ~ 0 V	100 μA
Pt/Ag nanodots/HfO_2_/Pt [22]	~ 10^9^	~ 10^9^	~ 0.25 V, ~ 0.05 V	1 mA
Ag/TaO_x_/TaO_y_/TaO_x_/Ag [21]	~ 10^10^	~ 10^10^	0.1–0.18 V, ~ 0 V	1 mA

**Fig. 4:**
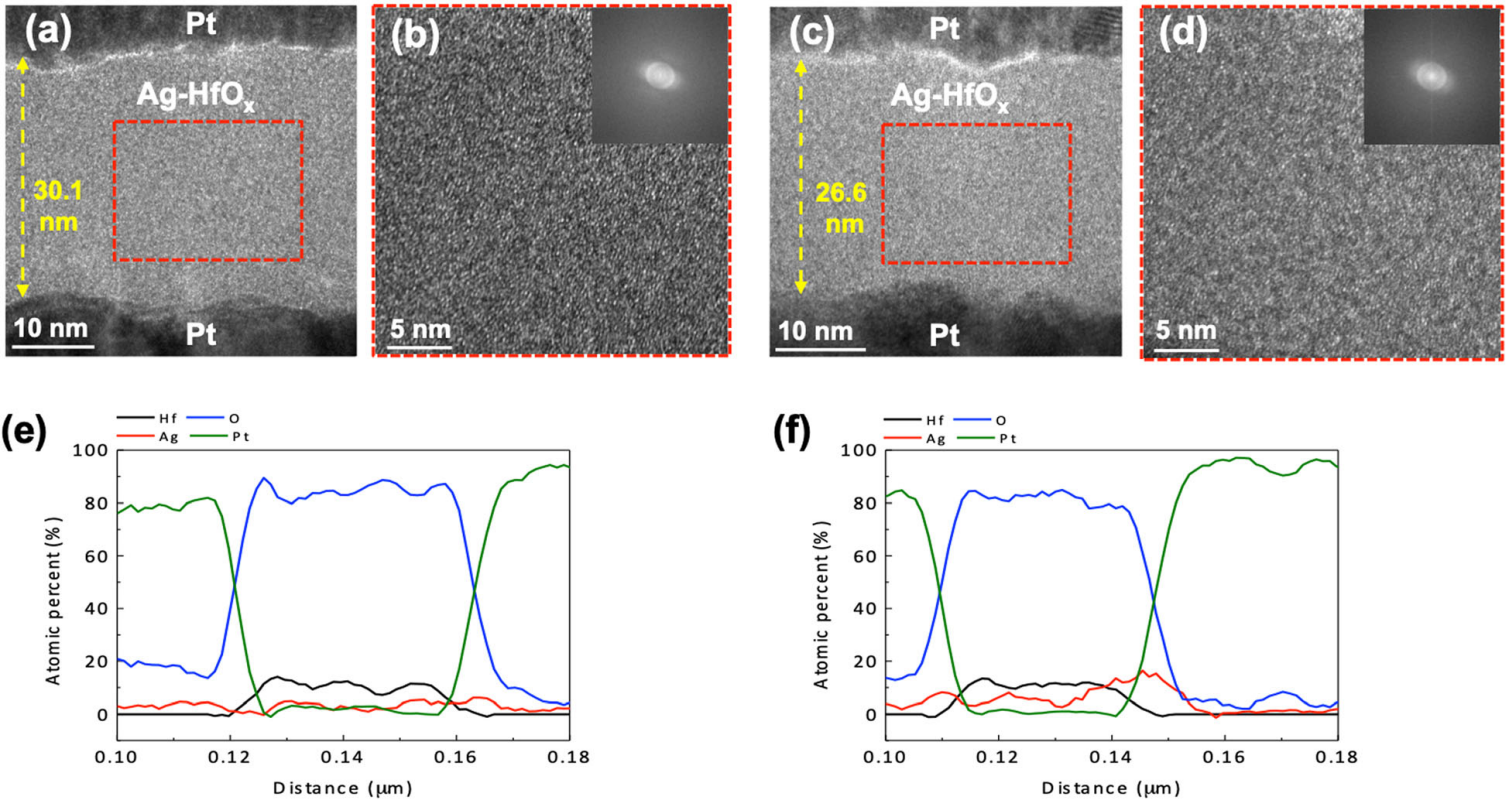
Microstructural and compositional analysis. **a** TEM cross-sectional image of D2. **b** Enlarged TEM image of D2. The inset is the corresponding FFT image. **c** TEM cross-sectional image of D3. **d** Enlarged TEM image of D3. The inset is the corresponding FFT image. EDS elemental line profiles of **e** D2 and **f** D3

To observe the size and distribution of the Ag atoms within the HfO_x_ layer, HR-TEM and EDS elemental analyses were performed on D2 and D3. Figure 4 a and b (c and d) show cross-sectional HR-TEM images of D2 (D3). According to the HR-TEM images, it is likely that the Ag atomic size is uniformly dispersed into the HfO_x_ matrix. No distinguishable Ag particles or Ag clusters a few nanometers in size were observed in both devices. In addition, the HfO_x_ amorphous phase was confirmed via a fast Fourier transform (FFT) image as shown in the insets of Fig. 4 b and d. However, Ag signal in the line profiles indicates Ag presence in the HfO_x_ layer. Thus, it is concluded that Ag in HfO_x_ would be distributed in atomic scale. The switching region including the Ag nanofilament should be investigated; however, the TS characteristics are volatile such that in situ TEM observation should be attempted in the future.

**Fig. 5:**
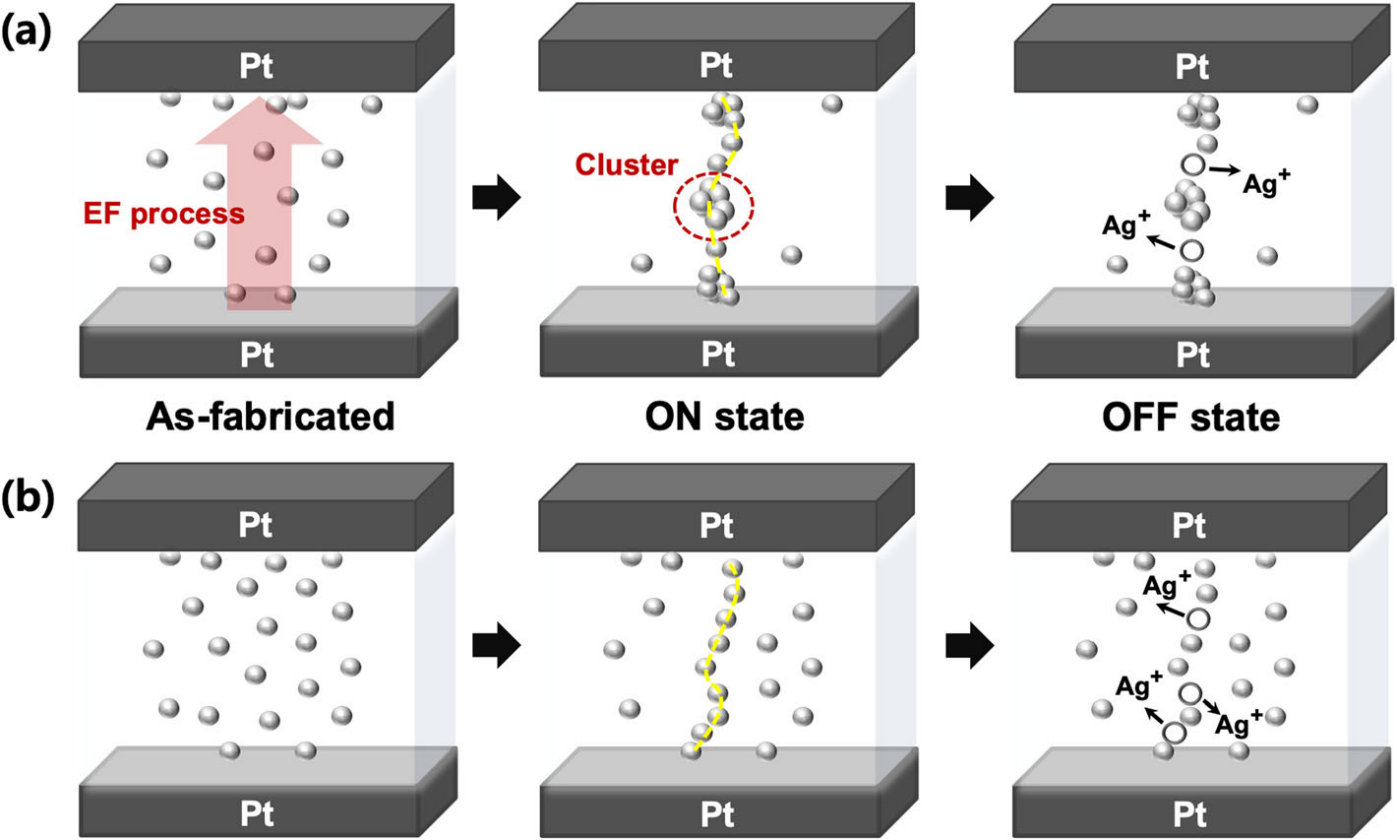
Suggested mechanism of TS in an Ag-doped HfOx device. The first Ag filament formation/rupture for TS behavior in **a** EF-needed (D2) and **b** EF-free (D3) devices

We propose the following mechanism for TS behaviors in our devices as shown in Fig. 5 a and b. The as-fabricated Ag-doped HfO_x_ switching layers have uniformly distributed Ag atoms in the HfO_x_. However, it is expected that there is a relatively long distance between the Ag atoms because of the low Ag concentration in D2. Figure 5a shows the EF process from as-fabricated D2. When an electric field is applied to the device in a pristine state, Ag atoms in the HfO_x_ can be oxidized into Ag^+^ ions and they migrate along the field direction. The oxidized Ag^+^ ions are reduced to Ag atoms again at the other Pt electrode, where an Ag-conducting filament continuously can grow. Once the filament is connected between the two electrodes, the device is switched to an ON state from an OFF state. During the EF process in D2, larger Ag clusters could be formed because of the high electric field. It was found that such a large electric field is sufficient to form Ag nanoparticles several nanometers in diameter from the in situ TEM observation in the literature [14, 15]. After the applied electric field is removed, Ag in atomic scale diffuse out into the HfO_x_ matrix, indicating that the device is returned to the OFF state. However, larger Ag clusters, which cannot sufficiently diffuse out, stay at conductive path. Thus, this residual Ag clusters lead to a larger OFF current in subsequent OFF state. In contrast, in the case of as-fabricated D3 as shown in Fig. 5b, Ag filament is formed without formation of Ag cluster because D3 is operated under the low electric field, meaning that the device can maintain low OFF current. Likewise, when the applied electric field is removed, the device is returned to the OFF state because of the spontaneous rupture of Ag filament that can be explained by the Thomson–Gibbs effect of minimizing the interfacial energy between a filament and matrix [15, 18, 28]. Consequently, bidirectional TS characteristics can be attained through repeatable Ag atom/ion diffusive dynamics.

To explain the difference during the EF process, we propose the following Eqs. (3) and (4):

3 $$ D={D}_0\mathit{\exp}\left(-\frac{E_{\mathrm{mo}}}{kT}\right) $$

where *D* is the diffusivity, *D*_0_ is the pre-exponential factor, *E*_mo_ is the migration barrier at zero bias, *k* is Boltzmann’s constant, and *T* is the local absolute temperature. To form the Ag filament within the HfO_x_ layer, Ag ions must overcome a migration barrier to move toward the negatively biased electrode. When the external bias is applied, the electric field can lower the migration barrier, *E*_m_, leading to ion migrations along the field direction as follows:

4 $$ {E}_{\mathrm{m}}={E}_{\mathrm{m}\mathrm{o}}-\frac{e\bullet {V}_{\mathrm{bias}}}{Z_{\mathrm{box}}}\bullet \Delta  z $$

with an electron charge *e*, bias voltage *V*_bias_, HfO_x_ layer thickness *Z*_box_, and Ag hopping distance in the *z* direction *∆z*. Indeed, Ag ions can hop in all directions under zero bias. However, we considered the hopping along the *z* direction in the EF process because the device can be turned to ON state under the high electric field where Ag ions dominantly hop along the electric field direction. To estimate the *E*_m_, we calculated the Ag hopping distance (*∆z*) as 0.67 nm in D2 and 0.45 nm in D3 from our experimental results of Ag concentration and film thickness. The migration barrier at zero bias, *E*_mo_ = 3.02 eV, was used from the literature [29]. In the first formation of the Ag filament, ~ 1.6 MV/cm of electric field was required in D2 and it lowered the migration barrier by 0.11 eV. In contrast, ~ 0.4 MV/cm of electric field was required for the first switching in D3 and it lowered the migration barrier by 0.04 eV. Thus, the Ag diffusion is sufficient to form the Ag filament in D3 despite the lower barrier reduction because of the short hopping distance and high Ag concentration compared with those of D2. However, because of the relatively long hopping distance in D2, the larger barrier reduction was needed for sufficient diffusion to form Ag filament. Once the filament forms, it should be spontaneously ruptured by ceasing the voltage; however, the Ag filament could not entirely diffuse out into their initial distribution, and thus the hopping distance *∆z* decreases from that of the as-fabricated D2. Therefore, this result led to a reduced electric field (~ 0.4 MV/cm) in subsequent threshold switching. It should be noted that film thickness considerably affects the *I*-*V* characteristics of the device. Therefore, we confirmed the EF process in the devices with similar thickness but having a different Ag concentration. Likewise, the devices exhibited a transition from the EF-needed to EF-free characteristics as the increase of Ag concentration. Therefore, it was demonstrated that Ag concentration essentially affects the EF process by controlling the effective internal electric field. That is, modulating the Ag concentration and thus the hopping distance in the HfO_x_ layer is required for EF-free TS characteristics with larger *NL* and *S* values.

## Conclusions

Diffusive selector devices based on Ag-doped HfO_x_ thin films were fabricated and their TS characteristics were evaluated. To understand the effect of the Ag concentration on the electrical properties, devices with different Ag concentrations were assessed. TS behavior in the devices can be described by the formation/self-rupture of Ag filament from atomically dispersed Ag atoms in the HfO_x_. It was confirmed that the Ag concentration could affect the EF process to form such a metallic filament. The device with a low Ag concentration required a precedent EF process for TS behavior, while EF-free TS behavior was proven in the device with a higher Ag concentration. In addition, the EF-free device showed better TS performance than that of the EF-needed device in terms of nonlinearity and OFF current. Therefore, proper dopant concentration and distribution control are required to obtain an EF-free diffusive selector device to prevent performance degradation resulting from the EF process.

## Supplementary information

**Additional file 1: Figure S1**. The surface morphology of Ag-doped HfOx devices. a)-d) exhibit AFM images in each device. RMS roughness is 0.29, 1.59, 2.15 and 3.55 nm, respectively. **Figure S2**. The top-view images of Ag-doped HfOxdevices. a)-d) exhibit FESEM images in each device.

## Data Availability

All data are fully available without restriction.

## Abbreviations

CBA: Crossbar array
EF: Electroforming
HRS: High-resistance state
LRS: Low-resistance state
*NL*: Nonlinearity
*S*: Selectivity
TS: Threshold switching

## References

[1] Jeong DS, Kim KM, Kim S, Choi BJ, Hwang CS (2016). Memristors for energy-efficient new computing paradigms. Adv Electron Mater.

[2] Zidan MA, Strachan JP, Lu WD (2018). The future of electronics based on memristive systems. Nat Electron.

[3] Xia Q, Yang JJ (2019). Memristive crossbar arrays for brain-inspired computing. Nat Mater.

[4] Torrezan AC, Strachan JP, Medeiros-Ribeiro G, Williams RS (2011). Sub-nanosecond switching of a tantalum oxide memristor. Nanotechnology.

[5] Lee MJ, Lee CB, Lee D, Lee SR, Chang M, Hur JH, Kim YB, Kim CJ, Seo DH, Seo S, Chung UI, Yoo IK, Kim K (2011). A fast, high-endurance and scalable non-volatile memory device made from asymmetric Ta2O5-xx/TaO2-xbilayer structures. Nat Mater.

[6] Pi S, Li C, Jiang H, Xia W, Xin H, Yang JJ, Xia Q (2018). Memristor crossbar arrays with 6-nm half-pitch and 2-nm critical dimension. Nat Nanotechnol.

[7] Li C, Hu M, Li Y, Jiang H, Ge N, Montgomery E, Zhang J, Song W, Dávila N, Graves CE, Li Z, Strachan JP, Lin P, Wang Z, Barnell M, Wu Q, Williams RS (2017). Analogue signal and image processing with large memristor crossbars. Nat Electron.

[8] Kim Y, Jeong WH, Tran SB, Woo HC, Kim J, Hwang CS, Min KS, Choi BJ (2019). Memristor crossbar array for binarized neural networks. AIP Adv.

[9] Cortese S, Khiat A, Carta D, Light ME, Prodromakis T (2016). An amorphous titanium dioxide metal insulator metal selector device for resistive random access memory crossbar arrays with tunable voltage margin. Appl Phys Lett.

[10] Son M, Lee J, Park J, Shin J, Choi G, Jung S, Lee W, Kim S, Park S, Hwang H (2011). Excellent selector characteristics of nanoscale VO2 for high-density bipolar ReRAM applications. IEEE Electron Device Lett.

[11] Aluguri R, Tseng T-Y (2016). Overview of selector devices for 3-D stackable cross point RRAM arrays. J Electron Device Soc.

[12] Gao T, Feng J, Ma H, Zhu X, Ma Z (2019). Al x Te 1-x selector with high ovonic threshold switching performance for memory crossbar arrays. Appl Phys Lett.

[13] Choi BJ, Zhang J, Norris K, Gibson G, Kim KM, Jackson W, Zhang MXM, Li Z, Yang JJ, Williams RS (2016). Trilayer tunnel selectors for memristor memory cells. Adv Mater.

[14] Wang Z, Joshi S, Savel’ev SE, Jiang H, Midya R, Lin P, Hu M, Ge N, Strachan JP, Li Z, Wu Q, Barnell M, Li GL, Xin HL, Williams RS, Xia Q, Yang JJ (2016). Memristors with diffusive dynamics as synaptic emulators for neuromorphic computing. Nat Mater.

[15] Wang Z, Rao M, Midya R, Joshi S, Jiang H, Lin P, Song W, Asapu S, Zhuo Y, Li C, Wu H, Xia Q, Yang JJ (2018). Threshold switching of Ag or Cu in dielectrics: materials, mechanism, and applications. Adv Funct Mater.

[16] Song J, Prakash A, Lee D, Woo J, Cha E, Lee S, Hwang H (2015). Bidirectional threshold switching in engineered multilayer (Cu2O/Ag:Cu2O/Cu2O) stack for cross-point selector application. Appl Phys Lett.

[17] Bin HU, Lee D, Lee JS (2017). Reliable current changes with selectivity ratio above 109 observed in lightly doped zinc oxide films. NPG Asia Mater.

[18] Midya R, Wang Z, Zhang J, Savel’ev SE, Li C, Rao M, Jang MH, Joshi S, Jiang H, Lin P, Norris K, Ge N, Wu Q, Barnell M, Li Z, Xin HL, Williams RS (2017). Anatomy of Ag/Hafnia-based selectors with 1010 nonlinearity. Adv Mater.

[19] Yoon JH, Wang Z, Kim KM, Wu H, Ravichandran V, Xia Q, Hwang CS, Yang JJ (2018). An artificial nociceptor based on a diffusive memristor. Nat Commun.

[20] Sun J, Wang H, Song F, Wang Z, Dang B, Yang M, Gao H, Ma X, Hao Y (2018). Physically transient threshold switching device based on magnesium oxide for security application. Small.

[21] Sun Y, Zhao X, Song C, Xu K, Xi Y, Yin J, Wang Z, Zhou X, Chen X, Shi G, Lv H, Liu Q, Zeng F, Zhong X, Wu H, Liu M, Pan F (2019). Performance-enhancing selector via symmetrical multilayer design. Adv Funct Mater.

[22] Hua Q, Wu H, Gao B, Zhao M, Li Y, Li X, Hou X, Marvin Chang MF, Zhou P, Qian H (2019). A threshold switching selector based on highly ordered Ag nanodots for X-point memory applications. Adv Sci.

[23] Yoo J, Woo J, Song J, Hwang H (2015). Threshold switching behavior of Ag-Si based selector device and hydrogen doping effect on its characteristics. AIP Adv.

[24] Song J, Woo J, Prakash A, Lee D, Hwang H (2015). Threshold selector with high selectivity and steep slope for cross-point memory array. IEEE Electron Device Lett.

[25] Sun H, Liu Q, Li C, Long S, Lv H, Bi C, Huo Z, Li L, Liu M (2014). Direct observation of conversion between threshold switching and memory switching induced by conductive filament morphology. Adv Funct Mater.

[26] Chae BG, Seol JB, Song JH, Baek K, Oh SH, Hwang H, Park CG (2017). Nanometer-scale phase transformation determines threshold and memory switching mechanism. Adv Mater.

[27] Sun H, Liu Q, Li C, Long S, Lv H, Bi C, Huo Z, Li L, Liu M (2014). Direct observation of conversion between threshold switching and memory switching induced by conductive filament morphology. Adv Funct Mater.

[28] Valov I, Linn E, Tappertzhofen S, Schmelzer S, Van Den Hurk J, Lentz F, Waser R (2013). Nanobatteries in redox-based resistive switches require extension of memristor theory. Nat Commun.

[29] Dai YH, Chen Z, Jin B, Li N, Li XF (2015). Optimal migration path of Ag in HfO 2 : a first-principles study. Chinese Phys B.

