# Supplementary material for: Effect of Ag Concentration Dispersed in HfOx Thin Films on Threshold Switching
Source: Nanoscale Res Lett. 2020 Jan 30;15:27. doi: 10.1186/s11671-020-3258-6 (PMC6990205; doi:10.1186/s11671-020-3258-6)
Supplement: Supplementary file 1 — Additional file 1: Figure S1. The surface morphology of Ag-doped HfOx devices. a)-d) exhibit AFM images in each device. RMS roughness is 0.29, 1.59, 2.15 and 3.55 nm, respectively. Figure S2. The top-view images of Ag-doped HfOxdevices. a)-d) exhibit FESEM images in each device. [file 11671_2020_3258_MOESM1_ESM.pdf]

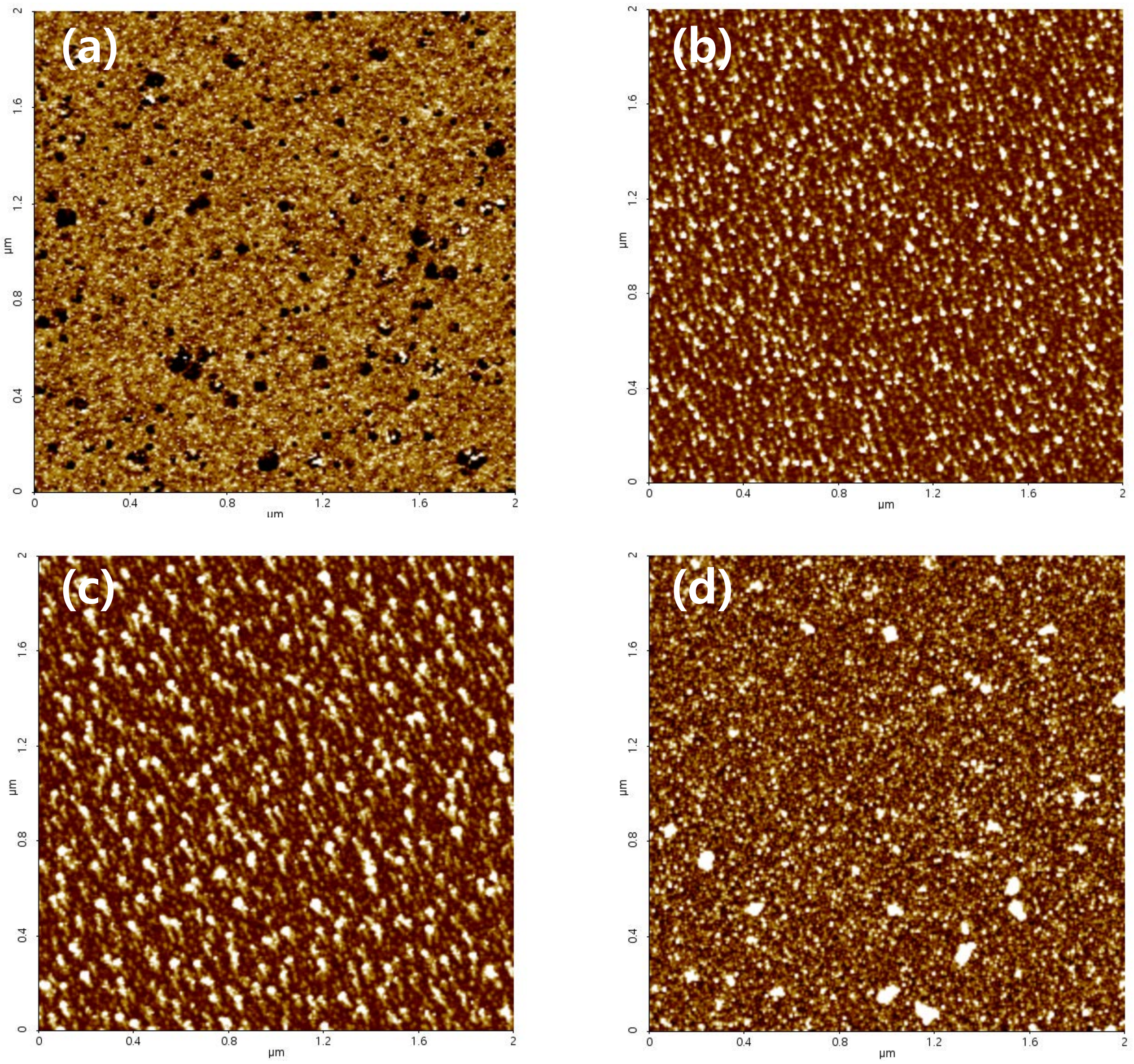

Figure S1. The surface morphology of Ag-doped HfO_x devices. a)-d) exhibit AFM images in each device. RMS roughness is 0.29, 1.59, 2.15 and 3.55 nm, respectively.

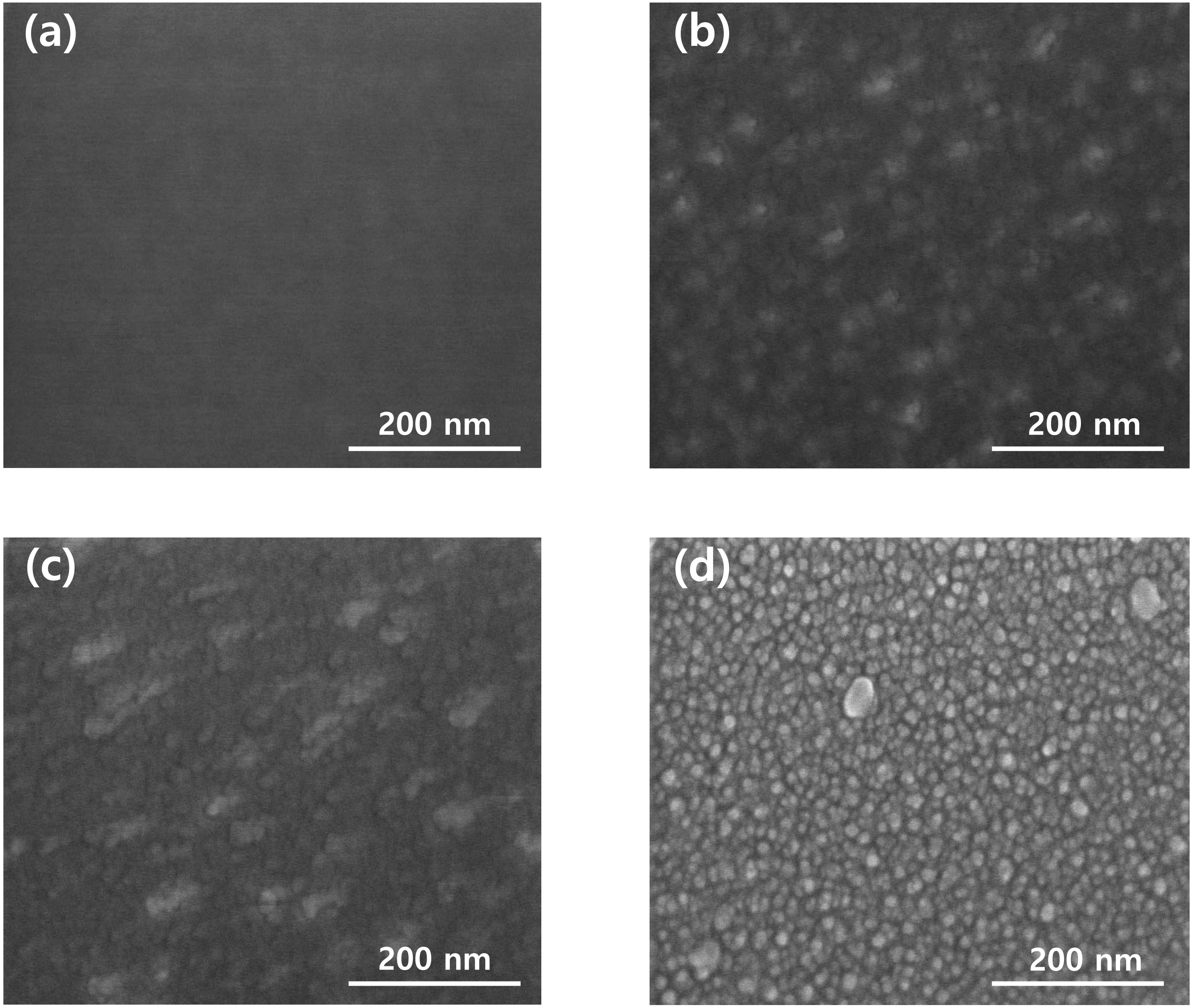

Figure S2. The top-view images of Ag-doped HfO_x devices. a)-d) exhibit FESEM images in each device.
